# Comparative Study on Chemical Constituents of Ginseng Flowers with Four Consecutive Cultivation Age

**DOI:** 10.1155/2023/1771563

**Published:** 2023-04-04

**Authors:** Lian-Yun Du, Hui-E. Zhang, Ye Zhang, Yan-Yan Han, Ping Ye, Xiang-Ru Meng, Yan-Long Shen, Chang-Bao Chen, Mei-Ling Fan, En-Peng Wang

**Affiliations:** ^1^Jilin Ginseng Academy, Changchun University of Chinese Medicine, Changchun 130117, Jilin, China; ^2^State Key Laboratory of Supramolecular Structure and Materials, College of Chemistry, Jilin University, Changchun 130012, Jilin, China; ^3^Affiliated Hospital of Changchun University of Chinese Medicine, Changchun 130117, Jilin, China

## Abstract

The harvest period of cultivated ginseng is generally 4–6 years. Ginseng flowers (GFs), the nonmedicinal parts, are usually removed every autumn, in which components are generally believed to stay unchanged with the increasing cultivation age. Recently, few documents were reported on the variation of volatile organic compounds (VOCs) and other components about ginseng flowers. This study had an insight into the variation of the chemical constituents with the cultivation ages through the comparison of the volatile organic compounds, gross ginsenosides, crude polysaccharide, and gross proteins of ginseng flowers from 3-, 4-, 5-, and 6-yr-old (GF3, GF4, GF5, and GF6) which were conducted by headspace solid-phase microextraction-gas chromatography-triple quadrupole mass spectrometry (HS-SPME-GC-QQQ/MS) and spectroscopic analysis combined with multivariate statistical analysis, including one-way ANOVA analysis and *T* test. The results indicated that the crude polysaccharide contents raised significantly depending on cultivation age except 6-yr-old, whereas the gross ginsenosides and the gross protein content were indistinctive. According to the peak intensity of determined VOCs, the contents of most differential compounds arranged in an order from high to low are GF3, GF4, GF5, and GF6, such as the compounds **2**–**15**, **17**–**19**, **22**, and **25**–**26**, therefore, they can be inferred that they are important markers to identify the age of GFs. 461 common differential compounds were gained and 26 common volatile organic compounds were identified with RSI >800 and RI and RIx no more than 30, including alcohols (such as **11**, **12**, and **15**), sesquiterpenes (such as **2**, **3**, and **4**), esters (such as **1** and **26**), naphthalene and naphthol (such as **7** and **20**), which had potential effects on curing Alzheimer's disease, inflammatory diseases, and prostate cancer based on network pharmacology analysis. This paper firstly revealed the variation rules of constitutions of GFs, which may provide a reference for the harvest and making rational application.

## 1. Introduction

Ginseng (*Panax ginseng* C. A. Mey.) is a kind of herb belonging to the Araliaceae family with the reputation of being “The King of Herbs” that has a long international history [1]. The root and rhizome, the stem, leaf, flower bud, and berry of the ginseng are rich in ginsenosides [2–4]. Recently, interest in the flower buds, as a component of ginseng, has grown because they have valuable medicinal potential with antifatigue and immunity-enhancing functions [5], although ginseng flowers (GFs) buds are not recorded in the Chinese Pharmacopoeia (2020 edition) [6]. It has been reported that ginsenosides and polysaccharide are the major bioactive constituents of GFs with gross ginsenoside contents approximately five times than those in the ginseng root [7–9].

The quality of traditional Chinese medicinal (TCM) materials is usually defined by their texture and color but their VOCs have rarely been considered. VOCs acting as sensory indicators or a marker play a key role in production or evaluating product quality because of the potent efficacy of VOCs [10]. They represent about 1% of plant secondary metabolites with around 1700 substances, mediate plant semi chemistry, and are involved in abiotic stress responses [11]. Extraction is a crucial procedure for analyzing VOCs with many methods being reported, such as hydro-distillation, microwave-assisted extractions, and carbon dioxide supercritical fluid extraction [12, 13]. However, these methods are time- and labor-consuming. Recently, headspace solid-phase microextraction (HS-SPME) has been increasingly used for analyzing VOCs, researchers can perform reliable analysis of VOCs of different kind of samples, especially aldehydes, ketones, alcohols, esters, phenolic compounds, and some key terpenic hydrocarbons, such as foodstuffs, plants, and their metabolites, because the technique is generally solvent-free, quite rapid, require minimum sample preparation, and minimize the matrix constituents [14–16].

Chemometrics is a discipline related to the application of mathematics, statistics, and computer science. The main missions of the chemometrics are manipulating and analyzing the chemical data, dealt with complex data sets to obtain the chemical information to the maximum extent [17-18]. At present, an increasing number of applications of chemometric pattern recognition in the analysis of food or agricultural products, such as wine aging studies, authenticity identification of TCM, and evaluation of the extraction method of artichoke [19–21].

The present study will use physicochemical methods to determine the content of gross ginsenosides, crude polysaccharide, and gross protein in GFs from 3-, 4-, 5-, and 6-yr-old ginseng plants (GF3, GF4, GF5, and GF6), which will be extracted and analyzed by the headspace solid-phase microextraction-gas chromatography-mass spectrometry (HS-SPME-GC-QQQ/MS). Pattern recognition approaches, such as principal component analysis (PCA) and orthogonal partial least squares-discriminant analysis (OPLS-DA), will be used to discover potential chemical markers. The results will be useful for clearly discriminating between GFs of various ages, and thus improve the quality control of GFs both as a raw drug material and for their use in TCM formulations.

## 2. Materials and Methods

### 2.1. Sample Collection, Chemicals, and Reagents

GFs of various ages were collected in June 2019 from Fusong (Jilin) and authenticated by Professor Changbao Chen (Changchun University of Chinese Medicine). After steaming at 40°C for 48 h, then crushed through 20-mesh sieves.

Ginsenoside Re (purity >98%) was obtained from the Department of Organic Chemistry, Jilin University; bovine serum albumin (BSA) was purchased from the Chinese Academy of Metrology; the glucose standard was purchased from Beijing Putian Tongchuang Biotechnology Co., Ltd. (Beijing, China); n-alkanes (C_8_-C_30_) was purchased from Alfa Aesar (Tewksbury, MA, USA); methanol, anhydrous ethanol, concentrated sulfuric acid, and glacial acetic acid (all analytically pure) were purchased from Beijing Chemical Plant (Beijing, China); polydimethylsiloxane-divinylbenzene solid-phase microextraction head (PDMS-DVB 65 *μ*m) and 12 mL headspace vials were purchased from Supelco (Bellefonte, PA, USA).

### 2.2. Contents of Gross Ginsenosides in GFs of Various Ages

 The gross ginsenosides content was determined by using the reference method of the State Standard of the People's Republic of China (GB/T 19506-2009): GF powder (passed through a 60-mesh sieve) was accurately weighed (about 1.0 g) then packaged in a neutral filter paper. The powder was extracted with ether using a Soxhlet extractor for 1 h. The sample package was then dried to evaporate the ether solvent. Methanol was added to the extractor to soak overnight. The next day, the appropriate amount of methanol was added to repeat the extraction for 6 times. The methanol extracts were combined and recovered, steaming a small amount of methanol extracts in the water bath and then dissolving them in water. 30 mL water extract was extracted for 4 times with 30 mL of water-saturated n-butanol. The upper liquid was steamed, then dissolved in methanol and made up to 10 mL. Finally, the end product was used as the ginsenosides sample solutions for further analysis.

The total ginsenoside content was determined using the reference method of the product of geographical indication-Jilin Changbaishan ginseng (GB/T 19506-2009), Ginsenoside Re (National Institutes for Food and Drug Control, Beijing, China)was used as the standard to calculate the content of the gross ginsenoside. In order to prepare the ginsenoside Re standard solution, 10 mg ginsenoside Re was put into a 10 mL volumetric flask, dilute to scale with methanol as solvent and mixed. 10 *μ*L, 20 *μ*L, 30 *μ*L, 40 *μ*L, 60 *μ*L, 80 *μ*L, and 100 *μ*L of the standard solution and 30 *μ*L sample solution were transferred to 10 mL tubes and dried (60°C water bath). Then, 0.5 ml 8% vanillin-ethanol and 5 ml 72% concentrated sulfuric acid were added to the prepared tubes. After fully shaking and mixing, the solution was heated in a 60°C water bath for 10 min and then cooled down in an ice-water bath for 10 min immediately. The mixed reagent without ginsenoside was used as a reference. Finally, both were determined at 544 nm using an enzyme calibration (Infinite M200 PRO, Tecan, Switzerland). To reduce the error in determining the gross ginsenosides content, three parallel extracts were obtained from the same origin of GF raw materials. The changes in gross ginsenosides content in GFs of various ages were then compared. The following equation was used to calculate the gross ginsenoside content:

Equation (1) of Cui et al. [22] was used to calculate the gross ginsenosides content:

(1) $$\begin{array}{c} X\left(\%\right)=m_{1}*\frac{\left(A_{2}/A_{1}\right)}{m_{2}}*\;100\%\text{,} \end{array}$$

where *X* is the gross ginsenosides content; *m*_1_ represents the weight of ginsenoside Re, *m*_2_ is the weight of GF powder, and *A*_1_ and *A*_2_ are the absorbances of the ginsenoside Re standard solution and sample solution, respectively.

### 2.3. Contents of Crude Polysaccharide in GFs of Various Ages

The crude polysaccharide content of the GFs was determined by a phenol-sulfuric acid method [23]. GF powder (2.00 g) was accurately weighed and added with 70 mL of water and extracted using a 100 mL volumetric flask for 30 min by ultrasonic extraction. The extract solution was placed in a 100°C water bath for extraction for 4 h and diluted to a 100 mL after cooled. The 5 mL solution was mixed with 15 mL, 5 mL, and 5 mL of ethanol solution and centrifuged at a speed of 10000 r/min for 10 min, respectively. The supernatant was discarded and the residue was dissolved in 100 mL volumetric flask with water. Take 2 mL solution in 25 mL graduated test tube with stopper and shake well after added 5% phenol solution 1.0 mL. Add 5 mL sulfuric acid solution quickly and also shake for 5 min. Placing it in boiling water bath for 20 min and then cooled to room temperature. The glucose standard solution 0, 0.1, 0.2, 0.4, 0.6, 0.8, 1.0, 1.2, 1.4, and 1.6 mL was accurately removed and the standard curve was made by the previous method. The mixed reagent without glucose was used as a reference. Finally, both were determined at 486 nm using an enzyme calibration. Three parallel extracts were obtained from the same origin of GF raw materials.

### 2.4. Contents of Gross Protein in GFs of Various Ages

Different volumes (0, 10, 20, 30, 40, 50, and 60 *μ*L) of the BSA standard solution (1 mg/mL) were transferred to an Eppendorf tube, then PBS buffer was added to make up to a volume of 150 *μ*L. After taking out 15 *μ*L and placing in another tube in turn, 285 *μ*L of Coomassie brilliant blue solution was added, mixed well then left at room temperature for 5–10 min. The solution was then transferred to a 96-well plate to measure the absorbance at 595 nm by an enzyme calibration. The standard curve was drawn with protein concentration (mg/15 *μ*L) as the abscissa and optical density as the ordinate. The reagent without BSA was used as a blank control. 15 *μ*L of GF extracts were used to determine the gross protein content of GFs in the above method. The content values were gained by three parallel samples (*n* = 3).

### 2.5. Analysis of VOCs in GFs of Various Ages

#### 2.5.1. Sample Pretreatment

The VOCs from the GFs were extracted by using HS-SPME. A sample of GFs about 100 mg was accurately weighed and transferred to a 12 mL headspace vial and then placed in a water bath at 70°C for 30 min. Thereafter, the VOCs were collected using a PDMS-DVB SPME fiber by exposing the fiber to the headspace of 2 cm above the sample for another 45 min under the same conditions. The fibers were activated before sampling according to the instructions. After this step, the SPME fiber was inserted directly into the injection port of the GC system for thermal desorption (30 s) in a splitless mode.

#### 2.5.2. Preparation of Quality Control and Blank Samples

The quality control (QC) samples were obtained by mixing the same amount of all the analyzed samples. The QC samples were processed as described previously. To ensure the stability and reproducibility of the experiment, QC samples were injected once every 6 to 8 GF samples.

To eliminate interference between the experimental environment and different samples, the blank sample was injected before the GF samples of various ages, that is, the above method was used without any powder. Blank sample were injected before each QC sample.

#### 2.5.3. Gas Chromatograph-Mass Spectrometer Conditions

The GC-QQQ/MS system comprised of a TRACE 1310 system and TSQ ENDURA mass spectrometer with electron impact (EI) ion source and Xcalibur data processing system and was purchased from Thermo Scientific (Waltham, MA, USA). The GC-QQQ/MS instrument was used to analyze the VOCs of the GFs with a DB-5MS silica capillary column (30 m × 0.25 mm, 0.25 *μ*m). The temperature of the injector was set at 250°C. Helium (99.999% purity) was used as the carrier gas at a flow rate of 1.0 mL/min and a split ratio of 1 : 30. The temperature sequence of the column was programmed as follows: an initial temperature of 50°C held for 2 min, increased at 10°C/min to 200°C then increased to 280°C at 5°C/min and held there for 10 min. The mass detector was equipped with EI, the ionization voltage was set at 70 eV in the 50–550 m/z scan range for the mass spectra collection, and the temperatures of the ion source and mass spectrum transmission line were 280°C.

#### 2.5.4. Qualitative and Quantitative Analysis

The VOCs of the GFs were identified as described by Choi et al. [24]. GC-QQQ/MS was used to determine the total ion chromatogram (TIC) of the VOCs of the GFs. The chromatogram was processed by automatic mass spectral deconvolution and identification system (AMDIS) software (https://www.amdis.net/) to eliminate interference and increase the accuracy of matching results, overlapping peaks were separated to purify the mass spectra. Finally, the detection results were qualitatively analyzed using the NIST spectrometry library combined with the temperature-programmed retention indices (PTRI). The mixture of a homologous series of n-alkane standards (C_8_–C_30_) dissolved in n-hexane, employed for auxiliary qualitative analysis, was analyzed under the same conditions as the samples. The RI of the samples was calculated by a linear heating formula, equation (2), with the chemical structure with the closest similarity to the mass spectrum and RI value being selected as the best identification result.

(2) $$\begin{array}{c} RI=100_{n\;}+\frac{100\left(t_{x}-t_{n}\right)}{t_{n+1}-t_{n}}\text{,} \end{array}$$

where *n* represents the number of carbon atoms, *t*_x_ represents the retention time of the analyzed compound, and *t*_*n*_ and *t*_*n* + 1_ represent the retention times of the outflow peaks of the n-alkanes with carbon numbers of *n* and *n* + 1, respectively. Otherwise, *t*_*n*_ < *t*_*x*_ < *t*_*n* + 1_.

The quantitative method adopted in this experiment is the peak area normalization method.

#### 2.5.5. Statistical Analysis

The GC-MS data were collected by Xcalibur software (version 2.2). XCMS Online (https://xcmsonline.scripps.edu/) was used to preprocess the GC-QQQ/MS data, using peak matching, peak alignment, and peak area normalization, leading to the data table containing the sample name, retention time, mass-to-charge ratio, and peak intensity. The data table was then imported into SIMCA-p software (version 17.0, Umetrics AB, Umeå, Sweden) for processing and a discriminant model for distinguishing between GFs of various ages was established using PCA and OPLS-DA. The data for the different VOCs were presented as means ± standard errors. In combination with the *T* test differential compounds were screened out based on the variable importance in projection (VIP) > 1 and *p* < 0.05. Based on the literature combined with the NIST 11 library, only differential compounds with a relative strength index (RSI) greater than 800 were recorded, at the same time, the experimental RIx, which do not differ from the reported RI values by more than 30, are considered an important indicator for identification. The figures were drawn using Origin 2021 (OriginLab Corp., Northampton, MA, USA), GraphPad Prism 7 (Graph Pad Software, San Diego, CA, USA), and Draw Venn diagram online (https://bioinformatics.psb.ugent.be/webtools/Venn/).

## 3. Results and Discussion

### 3.1. Gross Ginsenoside Content

**Figure 1:**
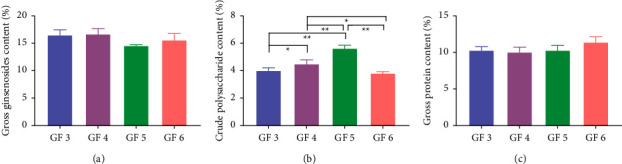
The content of gross ginsenosides. (a) Crude polysaccharide. (b) Gross protein. (c) In GFs of various ages. GF3, GF4, GF5, and GF6 represent ginseng flowers of 3-, 4-, 5-, and 6-yr-old, respectively, the symbols ^*∗*^ and ^*∗∗*^ are used to indicate statistical significance with *p* < 0.05 and *p* < 0.01, respectively.

The calibration curve and the gross ginsenosides content (external standard method) in GFs for each age were summarized in Tables 1 and 2, the method showed good linearity (*R*^2^ > 0.999).  Figure 1(a) shows that the gross ginsenoside content did not increase linearly. The GFs of a 4-yr-old exhibited the highest average content (16.68 ± 0.98%), and the 5-yr-old is the lowest (14.55 ± 0.20%), but the subsequent pairwise comparison models showed no significant differences (*p* > 0.05).

### 3.2. Crude Polysaccharide Content

The correlation coefficients between the content of crude polysaccharide in GFs are shown in Table 1. The specific values of the gross crude polysaccharide content (external standard method) in GFs for each age are summarized in Table 2, the method showed good linearity (*R*^2^ > 0.99).  Figure 1(b) shows that the crude polysaccharide content did not increase linearly, but tended to increase as the age increased from 3- to 5-yr-old and decrease as the age of 6-yr-old. The GF5 sample exhibited the highest average content (5.60 ± 0.25%) and the GF6 sample showed the lowest value (3.78 ± 0.14%), the pairwise comparison model showed differences (*p* < 0.05). Compared with GFs from 3-yr-olds, the crude polysaccharide content increased significantly at the 4-yr-old (*p* < 0.05) and extremely significant at the 5-yr-old (*p* < 0.01). Compared with those from 4-yr-olds, the content of GFs from 5-yr-olds increased extremely significant (*p* < 0.01) but 6-yr-old GFs decreased significantly (*p* < 0.05). Compared with GFs from 5-yr-olds, the crude polysaccharide content of GFs from 6-yr-olds had decreased extremely significant (*p* < 0.01).

### 3.3. Gross Protein Content

The calibration curve and the gross protein content (external standard method) in GFs for each age are summarized in Tables 1 and 2, the method showed good linearity (*R*^2^ > 0.99).  Figure 1(c) shows that the gross protein content did not increase linearly. The GF6 samples exhibited the highest average content (11.40 ± 0.73%) and the GF4 samples showed the lowest value (10.03 ± 0.67%), but the subsequent pairwise comparison models showed no significant differences (*p* > 0.05).

### 3.4. VOCs

#### 3.4.1. Multivariate Statistical Analysis of VOCs in GFs of Various Ages

Although the VOCs in the GFs of various ages can be measured by qualitative and quantitative analyses, these methods have little help in determining the characteristic VOCs in the samples. PCA is a multivariate statistical analysis method that can reduce the dimensionality of the data while information on the original data still retained by using several variables to select a smaller number of important variables by linearly transforming the data. Thus, PCA can reflect the trends in the data such as clusters and groupings with chemical similarities or differences [25–28], so this method can be used to determine the characteristic VOCs of GFs of various ages.

In the scatterplot of the PCA score (Figure S1), QC samples were clustered closely, indicating that the method was stable and reproducible. However, among the four groups of GFs of various ages, except that GF5-5 had poor clustering patterns in PCA models, the distribution effect of other samples was perfect.

#### 3.4.2. Discovering and Identifying Chemical Markers of VOCs in GFs of Various Ages

**Figure 2:**
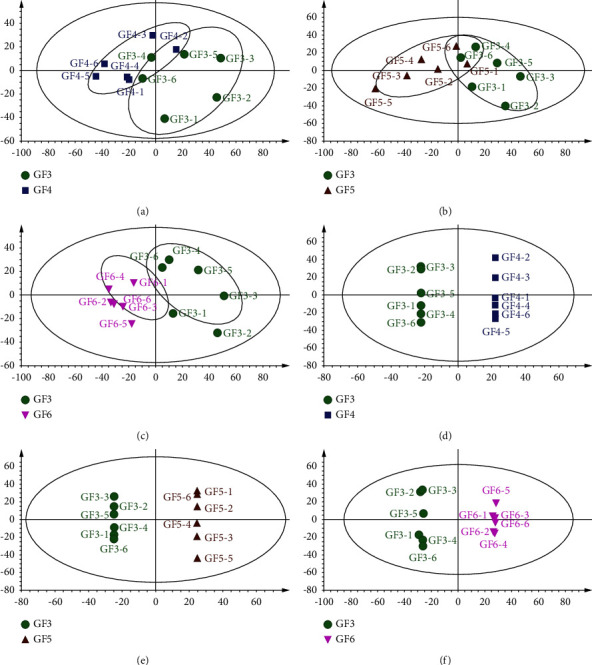
Score plots of PCA analysis (a)–(c). OPLS-DA analysis (d)–(f) of GF3 vs. GF4, GF3 vs. GF5, and GF3 vs. GF6. PCA: principal component; OPLS-DA: orthogonal partial least squares-discriminant analysis. For GF3 vs. GF4, *R*^2^*X* is 0.753, *R*^2^*Y* is 1, and *Q*^2^ is 0.795; for GF3 vs. GF5, *R*^2^*X* is 0.787, *R*^2^*Y* is 1, and *Q*^2^ is 0.924; for GF3 vs. GF4, *R*^2^*X* is 0.570, *R*^2^*Y* is 0.998, and *Q*^2^ is 0.892.

Supervised OPLS-DA was performed based on the above PCA model to define the most significant contributors towards discrimination. OPLS-DA not only extends PCA regression, but also provides better discriminatory ability than PCA on the larger divergence of intraclass for samples [29]. In the present study, the GF3 and GF4 samples (GF3 vs. GF4), GF3 and GF5 samples (GF3 vs. GF5), and GF3 and GF6 samples (GF3 vs. GF6) were distinctly separated in the PCA and OPLS-DA score plot (Figures 2(a)–2(f)), suggesting that the various ages contributed to the distinctive VOCs of the GFs. For example, the parameters value of *R*^2^*X* for the GF3 vs. GF4 of OPLS-DA model was 0.753, indicating that 75.3% of the variation in the dataset could be modeled by the selected components. The value of *R*^2^*Y* was 1, indicating that the model fitted the data very well. The value of *Q*^2^ was 0.795, indicating a good predictive ability. The other two groups of pairwise comparison models also exhibited perfect fitness and predictivity. Overall, establishing the OPLS-DA model was effective for discriminating between the GFs.

**Figure 3:**
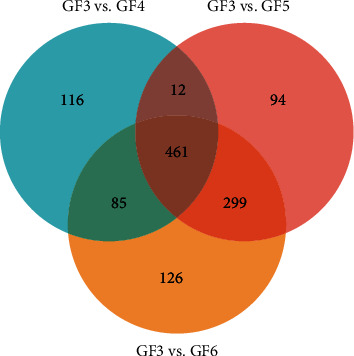
Venn diagram of the number of differential compounds of GF samples from 3-, 4-, 5-, 6-yr-old. 674 differential compounds were screened between GF3 vs. GF4, while 866 and 971 differential compounds between GF3 vs. GF5 and GF3 vs. GF6, respectively; Among them, there are 473 differential compounds in GF3 vs. GF4 and GF3 vs. GF5, 546 differential compounds in GF3 vs. GF4 and GF3 vs. GF6, and 760 differential compounds in GF3 vs. GF5 and GF3 vs. GF6.

**Figure 4:**
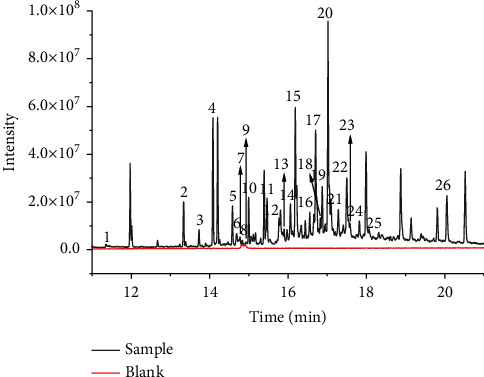
The total ion chromatogram (TIC) of the 26 identified common differential compounds. Number **1**–**26** represents 26 identified common differential compounds by NIST 11 in GFs.

**Figure 5:**
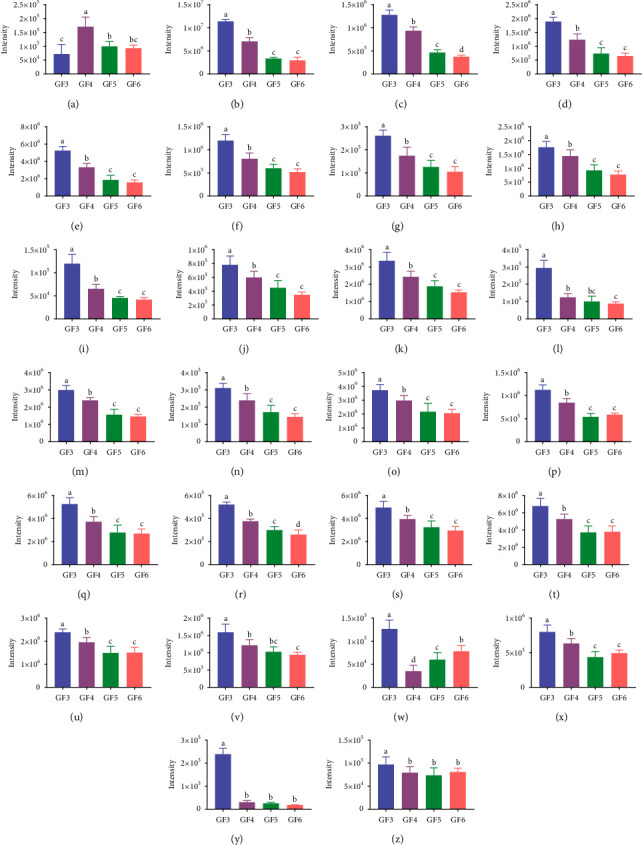
Profiles of 26 common differential metabolites from ginseng flower-revealing changes in intensities within various ages. Under Duncan's new multiple range test, different lowercase letters in the histogram by significant differences (*p* < 0.05) were determined.

**Figure 6:**
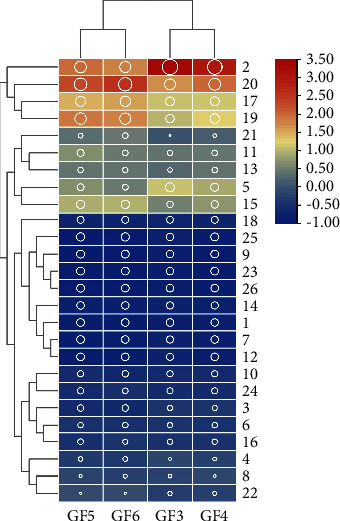
Heatmap and dendrogram of HCA of peak intensity of GFs of various ages. It is normalized for column scale; the size of the circle represents the size of the peak intensity; colors from red and yellow to blue represent the peak intensity of compounds from bigger to smaller.

Combined with the *T* test, the common differential compounds in GF3, GF4, GF5, and GF6 were screened out by the threshold of VIP > 1 and *p* < 0.05. 674 differential compounds were screened out between GF3 vs. GF4, 866 and 971 differential compounds were screened out between GF3 vs. GF5 and GF3 vs. GF6, respectively.  Figure 3 shows that 461 compounds with common symbolic differences in GFs from 4 ages were screened out. The TIC of the blank and 26 common differential compounds identified by NIST 11 and the literature are shown in Figure 4. It is not difficult to see that the blank had little effect on the sample. The preliminary list of components is shown in Table 3. The values of fold change describe the extent of the change from the initial to the final value. For the calculation of the fold change value of GFs of two ages, it was obtained by using the peak area of a compound of GF of the older age to that of the younger one. For example, the value of fold change was 2.3692, indicating that the peak intensity had increased by 2.3692 times between GFs from 3-yr-old to 4-yr-old.  Figure 5 summarizes the trend in the 26 identified common differential compounds of GFs and can also be visualized intuitively from the heatmap and dendrogram of HCA (Figure 6). For GFs of various ages, the colors of the heatmap and dendrogram varied dramatically in the GF3 and GF4 columns but varied more subtly in the GF5 and GF6 columns. This phenomenon indicated that depending on the age of the ginseng, the intensity of several peaks varied, thus demonstrating that different growth periods/ages change the metabolic composition of ginseng, the contents of GF3 and GF4 changed significantly, but over the next two years changed inapparently. Regarding specific compounds, it can be seen that the content of differential compound **1** was highest in GF4, and the contents of differential compounds **2**–**26** were highest in GF3. According to the peak intensity of determined VOCs, the contents of most differential compounds arranged in an order from high to low are GF3, GF4, GF5, and GF6, such as the compounds **2**–**15**, **17**–**19**, **22**, and **25**–**26**; therefore, they can be inferred that they are important markers to identify the age of GFs. But other differential compounds arranged in an order from high to low are GF3, GF4, GF6, and GF5, such as the differential compounds **16**, **20**–**21**, and **24**. It is notable that the lowest content of differential compound **23** was in GF4, followed by those from GF5, GF6, and GF3, showing a various trend from the other differential compounds.

### 3.5. Network Analysis between the Active Variable Metabolites and Diseases

**Figure 7:**
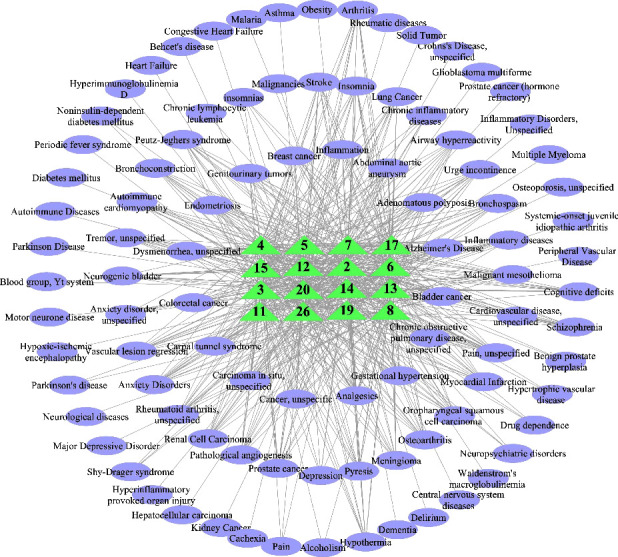
Network diagram based on 26 identified common differential compounds of VOCs and related diseases. The number including **2**, **3**, **4**, **5**, **6**, **7**, **8**, **11**, **12**, **13**, **14**, **15**, **17**, **19**, **20**, and **26**, indicating that the identified common differential compounds of VOCs in GFs of various ages have related diseases; green represents the differential compounds and purple represents the related diseases. Among them, alzheimer's disease, inflammatory diseases, and prostate cancer are the top 3 related diseases.

Recently, an increasingly interest of medical usage of GF which is leading to a rapid progress of research into their pharmacological effects on many related diseases and they are now considered to be a trustworthy plant with medicinal potential. With the increase of cultivation age, the main efficacy of GFs will change to a certain extent, which may be related to these 26 identified common differential compounds of VOCs. Accordingly, the 26 identified common differential compounds of VOCs are researched for the related diseases. The CAS, chemical name, or InChlKey of the 26 identified differential compounds were used to search in the TCMSP database.  Figure 7 shows that 95 diseases were related to 16 differential compounds retrieved from the TCMSP database, which were uploaded to Cytoscape software (version 3.8.2) for network mapping. Among them, 16 identified common differential compounds of VOCs found to be related to diseases are mainly sesquiterpenes and alcohols, including **2**, **3**, **4**, **5**, **6**, **7**, **8**, **11**, **12**, **13**, **14**, **15**, **17**, **19**, **20**, and **26**. For these diseases, Alzheimer's disease, inflammatory diseases, prostate cancer, breast cancer, anxiety disorders, insomnia, and lung cancer were the major diseases. By searching for common differential compounds and related diseases, we can predict the therapeutic effect of GFs of different ages on related diseases.

Polysaccharides and proteins, the basic substances concerned with plant growth and development, are involved in a series of physiological activities such as seed germination and development, root and leaf differentiation, fruit ripening, embryo formation, and senescence. The present study found that the crude polysaccharide content of GFs increased significantly at the age of 3 to 5, but decreased apparently at the age of 6, but there was no significant difference in the content between 3- and 6-yr-old, the results showed that the accumulation of crude polysaccharide of GFs was not positively correlated with the age of GFs. The gross ginsenosides and gross protein content of GFs changed slightly with the increase of age, but the changes were unobvious, indicating that the growth period of GFs had little effect on the gross ginsenosides and gross protein content.

The accumulation of chemical components is directly related to the activity of secondary metabolism-related regulatory enzymes and the expression of related genes. With the continuous growth and development of plants, the related enzyme activities and the expression level of regulatory genes will also change, resulting in the production and accumulation of chemical components in the process of plant growth and development are affected, therefore, the content of chemical components in different growth periods is different [30, 31]. Previous studies had shown that the content of volatile oil in flowers is affected by the distribution density of oil cells and the accumulation degree of volatile oil in oil cells [32]. Therefore, the content of VOCs in GFs may be closely related to the growth and development of oil cells. It can be inferred that ginseng blossoms after the 3-yr-old and the accumulation of early substances is deep, which can provide more energy and nutrition for the growth and development of oil cells in the buds of GFs. Therefore, the content of most VOCs is the highest in the 3-yr-old.

Sesquiterpenes and alcohols are the main identified common differential compounds [33]. Sesquiterpenes are an abundant group belonging to the terpenoid family, with a C15 structure comprise of three isoprene units. At least 300 types of sesquiterpenes have been discovered yet and numerous sesquiterpenes are an essential oil constituent which usually are released from the flowers and leaves as chemical messenger in plant signaling, particularly in the defense mechanism against biotic and abiotic stresses, such as to increase the plant tolerance, to inhibit the pathogen growth, and to attract pollinators or dispel predatory and parasitic insects [34]. Previous studies have shown that sesquiterpenes have anti-inflammatory and antioxidant effects, and the anti-inflammatory effect might mediate by the NF-*κ*B and MAPKs signaling pathways [35]. The results of the present study have shown that the age of the GF had an important influence on the chemical constituents and biological activities of GFs. For instance, if crude polysaccharide is the focus of research interest, it is recommended that GF5 should be harvested; if the target is on gross ginsenosides, gross proteins, or VOCs, it is advocated to harvest GF3.

## 4. Conclusions

GF is a potential medicinal part of ginseng and has been increasingly appreciated by consumers and growers worldwide. Elucidating the differences in the contents of GFs of various ages, the gross ginsenosides, crude polysaccharide, and gross protein were achieved using a physicochemical method. An accurate and feasible analytical method based on HS-SPME-GC-QQQ/MS combined with multivariate statistical analyses was then developed to allow a comprehensive comparison of the VOCs of GFs of various ages, such as alcohols, terpenes, esters, and so on, leading to establishing a discriminant model. To our knowledge, this is the first report that systematically compares the differences of the contents of 26 identified differential compounds in GFs of various ages. The method used in this investigation comprehensively analyzed the chemical composition of GFs, which can contribute to a reference for the collection and rational application of GFs of various ages, as well as can bring new methods and ideas for the identification and quality assessment of GFs of various ages and other Chinese medicinal material. However, this method still has some limitations, such as the nondestructive detection of ginseng age cannot be achieved. In the future, we hope to develop more techniques for nondestructive identification of ginseng age, which can provide theoretical guidance for future production practice and contribute to the development of ginseng industry.

## Figures and Tables

**Table 1:** The correlation coefficients between gross ginsenosides, crude polysaccharide, and gross protein contents of ginseng flowers (GFs).

Components	Calibration curves	*R* ^*2*^
Gross ginsenosides	*y* = 0.0019*x* − 0.0059	0.9991
Crude polysaccharide	*y* = 0.0040*x* + 0.0008	0.9988
Gross protein	*y* = 0.0058*x* + 0.0857	0.9978

**Table 2:** The contents of gross ginsenosides, crude polysaccharide, and gross protein in GFs of various ages (*n* = 3).

Components	GF3 (%)	GF4 (%)	GF5 (%)	GF6 (%)
Gross ginsenosides	16.51 ± 0.97	16.68 ± 0.98	14.55 ± 0.20	15.59 ± 1.20
Crude polysaccharide	3.96 ± 0.25	4.46 ± 0.33	5.60 ± 0.25	3.78 ± 0.14
Gross protein	10.30 ± 0.48	10.03 ± 0.67	10.28 ± 0.67	11.40 ± 0.73

**Table 3:** Identification of common differential compounds in GFs from 3-, 4-, 5-, and 6-yr-old.

Peaks	*t*/min	RI_x_	RI	RSI	Identification	GF4 vs. 3		GF5 vs. 3		GF6 vs. 3		CAS
						|log_2_ fold change|	Trends	|log_2_ fold change|	Trends	|log_2_ fold change|	Trends	
Esters												
1	11.04	1229	1225	845	3-Hexenyl-3-methylbutanoate	2.3692	Up	1.3944	Up	1.3045	Up	10032-11-8
26	20.08	1919	1919	821	Hexadecanoic acid, methyl ester	0.8165	Down	0.7553	Down	0.829	Down	112-39-0

Terpenes												
2	13.34	1393	1393	927	Cyclohexane,1-ethenyl-1-methyl-2,4-bis (1-methylethenyl)-, [1S-(1*α*,2*β*,4*β*)]-	0.6242	Down	0.2964	Down	0.2598	Down	515-13-9
3	13.73	1422	1423	897	Tricyclo [2.2.1.0 (2,6)] heptane,1,7-dimethyl-7-(4-methyl-3-pentenyl)-, (-)-	0.733	Down	0.3609	Down	0.2925	Down	512-61-8
4	14.08	1450	1450	924	(E)-*β*-Famesene	0.6548	Down	0.39	Down	0.3472	Down	18794-84-8
5	14.59	1489	1485	941	1,6-Cyclodecadiene,1-methyl-5-methylene-8-(1-methylethyl)-, [S-(E,E)]-	0.6304	Down	0.3595	Down	0.2948	Down	23986-74-5
6	14.69	1497	1497	896	Naphthalene,1,2,3,4,4a,5,6,8a-octahydro-4a,8-dimethyl-2-(1-methylethenyl)-, [2R-(2*α*,4a*α*,8a*β*)]-	0.676	Down	0.4982	Down	0.4338	Down	473-13-2
8	14.84	1509	1509	870	*β*-Bisabolene	0.8151	Down	0.5243	Down	0.4408	Down	495-61-4
9	14.93	1516	1513	836	(+)-Valencene	0.5445	Down	0.3795	Down	0.3467	Down	4630/7/3
10	15.01	1523	1531	822	trans-Z-*α*-bisabolene epoxide	0.7683	Down	0.5707	Down	0.4441	Down	-
14	16.07	1610	1612	845	Caryophyllene oxide	0.7711	Down	0.5562	Down	0.4656	Down	1139-30-6
16	16.58	1654	1646	801	Ledene oxide-(II)	0.7556	Down	0.4812	Down	0.522	Down	-
18	16.78	1671	1672	857	Aromadendrene oxide-(1)	0.7213	Down	0.576	Down	0.5022	Down	94020-95-8
19	16.88	1679	1678	839	Aromadendrene oxide-(2)	0.7956	Down	0.6549	Down	0.5982	Down	85710-39-0

Alcohols												
11	15.47	1561	1559	826	1,6,10-Dodecatrien-3-ol, 3,7,11-trimethyl-	0.7274	Down	0.5609	Down	0.4569	Down	7212-44-4
12	15.78	1586	1587	825	1H-cycloprop[e]azulen-4-ol, decahydro-1,1,4,7-tetramethyl-, [1aR-(1a*π*4*π*4a*π*7*π*7a*π*7b*π*]-	0.4204	Down	0.3405	Down	0.2994	Down	552-02-3
13	15.93	1598	1595	836	Ledol	0.7974	Down	0.5245	Down	0.4864	Down	577-27-5
15	16.2	1621	1619	933	(−)-Spathulenol	0.8015	Down	0.5889	Down	0.5577	Down	77171-55-2
17	16.74	1668	1668	882	*β*-santalol	0.705	Down	0.5284	Down	0.5149	Down	77-42-9
21	17.3	1714	1714	851	6-Isopropenyl-4,8a-dimethyl-1,2,3,5,6,7,8,8a-octahydro-naphthalen-2-ol	0.8203	Down	0.6263	Down	0.6352	Down	726134-57-2
22	17.52	1732	1732	820	2-(4a,8-Dimethyl-1,2,3,4,4a,5,6,7-octahydro-naphthalen-2-yl)-prop-2-en-1-ol	0.7628	Down	0.6464	Down	0.5929	Down	-
24	17.83	1757	1753	809	Lanceol, cis	0.7969	Down	0.5485	Down	0.6216	Down	10067-28-4
25	18.31	1795	1794	888	2-Hexadecanol	0.1308	Down	0.1057	Down	0.0787	Down	14852-31-4

Naphthalene and naphthol												
7	14.77	1503	1502	871	Naphthalene,1,2,4a,5,6,8a-hexahydro-4,7-dimethyl-1-(1-methylethyl)-	0.6686	Down	0.4835	Down	0.4011	Down	483-75-0
20	17.03	1692	1693	830	1-Naphthalenol,decahydro-1,4a-dimethyl-7-(1-methylethylidene)-, [1R-(1*α*,4a*β*,8a*α*)]-	0.7796	Down	0.5554	Down	0.5667	Down	473-04-1

Others												
23	17.55	1734	1739	828	Murolan-3,9(11)-diene-10-peroxy	0.2798	Down	0.4756	Down	0.6198	Down	-

## Data Availability

The data used to support the findings of this study are included within the article.

## Supplementary Materials

Supplementary Materials Supplementary Materials. Figure 1: score plots of principal component (PCA) analysis from metabolite profiling data of GC-MS of GF samples of various ages and QC samples.
