# Supplementary material for: Comparative Study on Chemical Constituents of Ginseng Flowers with Four Consecutive Cultivation Age
Source: Int J Anal Chem. 2023 Apr 4;2023:1771563. doi: 10.1155/2023/1771563 (PMC10089779; doi:10.1155/2023/1771563)
Supplement: Supplementary Materials — Figure 1: score plots of principal component (PCA) analysis from metabolite profiling data of GC-MS of GF samples of various ages and QC samples. [file 1771563.f1.docx]

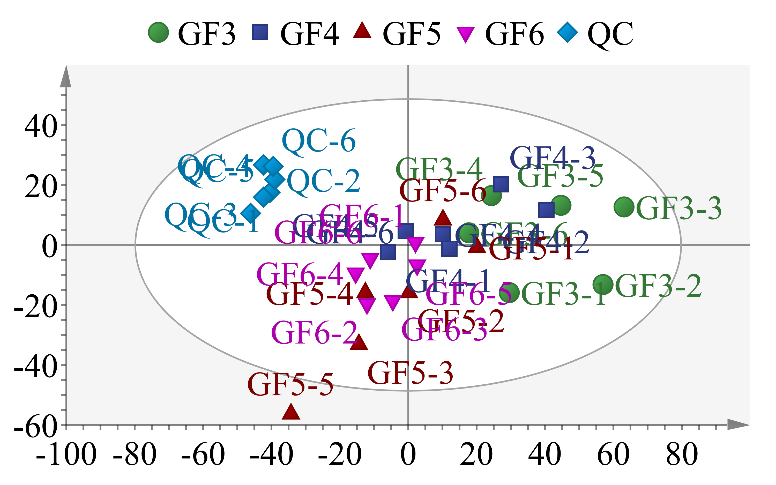


F_IGURE_ S1: Score plots of principal component (PCA) analysis from metabolite profiling data of GC-MS of GF samples of various ages and QC samples.
